# The Shelterin Component TPP1 Is a Binding Partner and Substrate for the Deubiquitinating Enzyme USP7*

**DOI:** 10.1074/jbc.M114.596056

**Published:** 2014-08-29

**Authors:** Ivo Zemp, Joachim Lingner

**Affiliations:** From the Swiss Institute for Experimental Cancer Research (ISREC), School of Life Sciences (SV), Ecole Polytechnique Fédérale de Lausanne (EPFL), 1015 Lausanne, Switzerland

**Keywords:** Deubiquitylation (Deubiquitination), Post-translational Modification (PTM), Shelterin, Telomerase, Telomere, TPP1, USP7

## Abstract

The telomeric shelterin component TPP1 has critical functions in telomeric protein complex assembly and telomerase recruitment and regulation. Here we identify USP7 as a novel interacting protein of the oligonucleotide/oligosaccharide-binding fold of TPP1, which was previously known to recruit telomerase to telomeres. We identify amino acids in TPP1 and USP7 that are critical for their interaction and multiple lysines within TPP1 that are oligo-ubiquitinated and deubiquitinated by USP7. Mutational analysis indicated that human TPP1 does not require ubiquitination for telomere association in contrast to previous observations reported for mouse Tpp1. Ubiquitination of human TPP1 also had no detectable effects on known protein interactions of TPP1 with TIN2, POT1, the CTC1-STN1-TEN1 complex, and telomerase. However, the close proximity of USP7 and telomerase binding sites on TPP1 suggest possible cross-talks. In addition, we found that TPP1 is degraded in a proteasome-dependent manner. Prevention of TPP1 ubiquitination prolonged TPP1 half-life ∼2-fold from 45 to 90 min, and remarkably, proteasome inhibition prompted complete stability of TPP1. This indicates that the proteasome destabilizes TPP1 through both direct and indirect pathways possibly involving TPP1-interacting proteins. Altogether, our work identifies novel regulatory circuits that contribute to TPP1 stability and function.

## Teaser

**Background:** Regulation of the telomeric protein TPP1 is critical for telomere function.

**Results:** TPP1 is ubiquitinated and is a substrate for the deubiquitinase USP7.

**Conclusion:** TPP1 ubiquitination does not affect its known protein interactions but leads to proteasome-dependent degradation.

**Significance:** These findings identify novel pathways that influence TPP1 stability and function.

## Introduction

The physical ends of linear eukaryotic chromosomes are formed by nucleoprotein structures termed telomeres. They protect chromosome ends from being recognized as DNA double strand breaks and therefore from nucleolytic processing and end-to-end fusions (1, 2). Semiconservative DNA replication is unable to fully copy the ends of linear chromosomes, leading to continuous shortening of the ends of telomeres with each round of DNA replication. In addition, nucleolytic end processing contributes to telomere shortening (3, 4). Upon reaching a critically short size, telomeres trigger a DNA damage response causing cellular senescence or apoptosis (5). This process limits the proliferative potential of normal human cells and therefore acts as a tumor suppression mechanism. To counteract telomere shortening, germ line and stem cells express the reverse transcriptase telomerase, which elongates telomeres by adding telomeric repeats to the 3′-end of the chromosome (6, 7).

Telomeric DNA is capped by the six-protein shelterin complex consisting of TRF1, TRF2, RAP1, TIN2, POT1, and TPP1 (8, 9). These proteins play crucial roles both in preventing the DNA damage response at telomeres and in regulating telomere elongation by telomerase (1, 10, 11). Within the shelterin complex, TPP1 is known to interact with both TIN2 and POT1 (12–14). These interactions are necessary for proper telomere association of POT1 and essential for telomere length regulation. Either depletion of TPP1 or disruption of the POT1-TPP1 interaction results in telomere elongation (12–15), indicating that TPP1 negatively controls telomerase. However, TPP1 also positively regulates telomerase. In a complex with POT1, TPP1 is able to stimulate telomerase activity and processivity (16, 17), and TPP1 was proposed to mediate telomerase recruitment to telomeres based on an interaction between TPP1, specifically its oligonucleotide/oligosaccharide-binding (OB)^3^ domain, and telomerase (15). Indeed, efficient telomerase recruitment in HeLa cells depends on TPP1 and its OB domain (18), and recent studies identified a surface region of the TPP1(OB) domain that mediates telomerase interaction and stimulation, the so-called TPP1 glutamine (E)- and leucine (L)-rich (TEL) patch (19–21). How the negative and positive roles of TPP1 in telomerase regulation are distinguished is not known to date. In addition, TPP1 might also play a role in further aspects of telomerase control, such as regulation of telomerase activity along the cell cycle or preferential elongation of short rather than long telomeres. It is tempting to speculate that post-translational modifications of TPP1 are involved in telomerase regulation, and indeed, recent findings suggest that phosphorylation of TPP1 may contribute to telomerase activation (22). Furthermore, studies in mouse have shown that RNF8-mediated ubiquitination of Tpp1 is required for retention of Tpp1 at telomeres (23).

Ubiquitination is a post-translational modification that is used to trigger proteasome-dependent protein degradation or to regulate protein function and localization. It is a reversible process with deubiquitinating enzymes reversing the action of E2/E3 ubiquitin ligases. In this study, we identified the deubiquitinase USP7 as an interacting protein of human TPP1 and show that TPP1 is ubiquitinated and is a substrate of USP7. In striking contrast to mouse Tpp1, TPP1 ubiquitination is not required for telomere localization in human cells. Moreover, TPP1 ubiquitination is neither required nor does it prevent the interactions of TPP1 with TIN2, POT1, telomerase, or the CTC1-STN1-TEN1 (CST) complex. However, TPP1 ubiquitination triggers proteasome-dependent degradation, suggesting that TPP1 levels may be tightly regulated.

## EXPERIMENTAL PROCEDURES

### 

#### 

##### Molecular Cloning

Plasmids encoding TPP1–3xFLAG, CTC1-V5, STN1-V5, and hTR have been described previously (18, 24, 25). Coding regions for TPP1 constructs were cloned into a pcDNA6-derived vector for expression as 3xHA-tagged proteins and into pLHCX for viral infections. Coding regions for USP7 constructs were amplified from pCI-neo Flag HAUSP (Addgene plasmid 16655) and cloned into pcDNA6-derived vectors for expression as mycHis-, GST-, or 3xFLAG-tagged proteins. Coding regions for TIN2, POT1, CTC1, STN1, and hTERT were cloned into pcDNA6-derived vectors for expression as 3xFLAG- or 13xmyc-tagged proteins, respectively. Site-directed mutagenesis of TPP1 or USP7 was performed using the QuikChange mutagenesis kit (Agilent Technologies). shRNA vectors for depletion of USP7 were prepared by cloning double-stranded DNA oligonucleotides into pSuper.puro. Target sequences used were: 5′-AGTCGTTCAGTCGTCGTAT (shUSP7_3) and 5′-CCAGCTAAGTATCAAAGGA (shUSP7_4).

##### Antibodies

Anti-HA-agarose (A2095) and anti-FLAG affinity gel (A2220) were obtained from Sigma. For Western blotting, secondary antibody against mouse (A21057) was purchased from Invitrogen, and secondary antibody against rabbit (611-732-127) was from Rockland. Antibodies against FLAG (F1804), tubulin (T9026), and His tag (H1029) were purchased from Sigma; anti-HA (MMS-101P) was from Covance; anti-USP7 (sc-30164) was from Santa Cruz Biotechnology; anti-myc (2276) was from Cell Signaling Technology; anti-V5 (46-0705) and secondary antibodies for immunofluorescence were from Invitrogen. Anti-TRF1 has been described previously (26).

##### Yeast Two-hybrid Screening

Yeast two-hybrid screening was carried out by Dualsystems Biotech AG, Zurich, Switzerland. The bait construct was generated by subcloning a cDNA encoding for amino acids 88–251 of TPP1 into pLexA-DIR (Dualsystems Biotech AG). The bait construct was transformed into strain NMY32 (*MATa his3*Δ*200 trp1-901 leu2-3*,*112* (*lexAop*)_8_-*ADE2 LYS2*::(*lexAop*)_4_-*HIS3 URA3*::(*lexAop*)_8_-*lacZ GAL4*) using standard procedures (27). Correct expression of the bait was verified by Western blotting of cell extracts. The absence of self-activation was verified by co-transformation of the bait together with a control prey and selection on minimal medium lacking the amino acids Trp, Leu, and His. For the yeast two-hybrid screen, the bait was co-transformed together with a normalized HeLa S3 library into NMY32. Positive transformants were tested for β-galactosidase activity using a pellet X-gal β-galactosidase assay (Dualsystems Biotech AG). Library plasmids were isolated from positive clones, and the identity of positive interactors was determined by sequencing.

##### Cell Transfections

Transient transfections of human embryonic kidney (HEK) 293T cells for co-immunoprecipitations or ubiquitination assays were performed using Lipofectamine 2000 (Invitrogen) according to the manufacturer's instructions. One to three wells of a 6-well plate were transfected per condition using 2.5 μg of total DNA and 10 μl of Lipofectamine/well. Equal amounts of the respective plasmids were used for co-transfections except for hTERT/hTR transfections for which hTR was kept in 5-fold excess over hTERT (and other plasmids). Transient transfections of HEK293T cells for purifications of ubiquitinated TPP1, TIN2–3xFLAG, POT1–3xFLAG, and CTC1–3xFLAG/STN1-V5 were carried out using the CalPhos transfection kit (Clontech, 631312) according to the manufacturer's instructions. Between three and 15 15-cm dishes were transfected per experiment using 50 μg total of DNA/dish.

To generate cell lines stably expressing TPP1–3xFLAG or -3xHA constructs, HEK293T cells were transfected with a 3:1:4 mixture of pMD-GAGPOL:pMD-VSG:pLHCX plasmids (3 wells per construct) using Lipofectamine. Cells were split 16 h post-transfection for expansion. 48 h post-transfection, virus-containing supernatants from the HEK293T cells were collected, filtered (0.2 μm) using a syringe, supplemented with Polybrene to a final concentration of 4 μg/ml, and added to HeLa cells at 50% confluence. 24 h postinfection, HeLa cells were split, and infected cells were selected by addition of hygromycin to a final concentration of 250 μg/ml for 7 days.

##### Co-immunoprecipitation Assays

For co-immunoprecipitations of TPP1 with telomerase, extracts were prepared 48 h post-transfection as described (19). The NaCl concentration was then adjusted to 300 mm, and extracts were incubated with anti-HA-agarose (Sigma) for 4 h. After three washes with 10 mm Tris/HCl, pH 8.0, 300 mm NaCl, 2 mm MgCl_2_, beads were eluted with SDS-PAGE sample buffer without reducing agent for 5 min at 60 °C. DTT was added after the elution to a final concentration of 50 mm.

For all other co-immunoprecipitations, cells were detached using PBS containing 0.5 mm EDTA and centrifuged (400 × *g* for 4 min at 4 °C). Cells were washed with cold PBS and centrifuged again. Cell pellets were resuspended in 10 mm Tris/HCl, pH 8.0, 150 mm NaCl, 2 mm MgCl_2_, 0.5% Nonidet P-40 supplemented with protease inhibitor mixture (Sigma). After incubation on ice for 30 min, samples were centrifuged for 10 min at 20,000 × *g* at 4 °C. Extracts corresponding to equal amounts of total protein (between 1.5 and 3 mg) were supplemented with cell lysis buffer to a volume of 400 μl and with 800 μl of 10 mm Tris/HCl, pH 8.0, 150 mm NaCl, 2 mm MgCl_2_ and incubated with 20 μl of anti-FLAG affinity gel, 20 μl of anti-HA-agarose, or 4 μg of anti-USP7 on a rotating wheel at 4 °C for 4 h. For USP7 immunoprecipitations, 20 μl of Protein G-Sepharose (GE Healthcare) was added after 2 h. After washing three times with the same buffer, beads were eluted with SDS-PAGE sample buffer without reducing agent for 4 min at room temperature. DTT was added after the elution to a final concentration of 50 mm.

##### Ubiquitination Assays

Cells were harvested 48 h after transfection of the reporter constructs directly or after treatment with 10 μm MG132 for 2 h. Cells were detached using PBS containing 0.5 mm EDTA, 2 mm *N*-ethylmaleimide and centrifuged (400 × *g* for 4 min at 4 °C). Cells were washed with cold PBS containing 2 mm *N*-ethylmaleimide and centrifuged again. Cell pellets were resuspended in 1 ml of urea lysis buffer (8 m urea, 100 mm sodium phosphate, pH 8.0, 15 mm imidazole) and sonicated with 25 pulses of a Branson Sonifier 250 tip sonicator. Samples were incubated on a rotating wheel for 10 min at room temperature and centrifuged for 10 min at 20,000 × *g* at 10 °C. Extract corresponding to equal amounts of total protein (between 2 and 4 mg) were incubated on a rotating wheel for 4 h at room temperature with 20 μl of nickel-nitriloacetic acid (Ni-NTA)-agarose beads. Beads were then washed twice with 6 m guanidine HCl, 100 mm sodium phosphate, pH 8.0, 0.1% Triton X-100, 15 mm imidazole; once with 8 m urea, 100 mm sodium phosphate, pH 8.0, 0.5% Triton X-100, 15 mm imidazole; and once with urea lysis buffer. Beads were eluted with 60 μl of urea lysis buffer containing 100 mm imidazole for 5 min at room temperature. SDS-PAGE sample buffer was added to the eluate.

##### Protein Purifications

For purification of ubiquitinated TPP1, HEK293T cells were harvested 48 h after transfection of reporter constructs and after 2 h of treatment with 10 μm MG132. Cells were harvested and lysed, and Ni-NTA binding was performed as described above in ubiquitination assays. For cells from one 15-cm dish, 1 ml of urea lysis buffer was used. Ubiquitinated proteins were bound on a column of 500 μl of Ni-NTA beads and eluted twice with 1.2 ml of urea lysis buffer containing 100 mm imidazole. The eluates were combined and rebuffered overnight to 25 mm Tris/HCl, pH 8.0, 150 mm NaCl, 2 mm MgCl_2_ using a Slide-A-Lyzer dialysis cassette (Thermo Scientific). Samples were centrifuged for 10 min at 20,000 × *g* at 4 °C to remove precipitated proteins, and supernatants were then incubated with 120 μl of anti-HA-agarose for 4 h on a rotating wheel at 4 °C. Beads were washed twice with 25 mm Tris/HCl, pH 8.0, 150 mm NaCl, 2 mm MgCl_2_ and twice with the same buffer containing 10% glycerol. Purified ubiquitinated TPP1 was stored on beads at −80 °C until use.

For purification of USP7, TIN2, POT1, and CTC1-STN1, HEK293T cells were harvested 48 h after transfection. Cells were detached using PBS containing 0.5 mm EDTA and centrifuged (400 × *g* for 4 min at 4 °C). Cells were washed with cold PBS and centrifuged again. Cell pellets were resuspended in 10 mm Tris/HCl, pH 8.0, 150 mm NaCl, 2 mm MgCl_2_, 0.5% Nonidet P-40 supplemented with protease inhibitor mixture (Sigma). Cells from one 15-cm dish were resuspended in 1 ml of lysis buffer. After incubation on ice for 30 min, samples were centrifuged for 10 min at 20,000 × *g* at 4 °C. The extract was diluted 2-fold with 25 mm Tris/HCl, pH 8.0, 150 mm NaCl, 2 mm MgCl_2_ and incubated with 200 μl of anti-FLAG affinity gel for 4 h on a rotating wheel at 4 °C. Beads were washed twice with 25 mm Tris/HCl, pH 8.0, 150 mm NaCl, 2 mm MgCl_2_ and once with 25 mm Tris/HCl, pH 8.0, 300 mm NaCl, 0.1% Triton X-100, 2 mm MgCl_2_, 10% glycerol. Proteins were then eluted by two incubations in the same buffer supplemented with 1 mg/ml 3xFLAG peptide (Sigma) for 15 min on a ThermoShaker (interval shaking at 1200 rpm at 37 °C). The eluate was filtered through a 35-μm Mobicol filter (MoBiTec), aliquoted, and stored at −80 °C until use. Purified protein fractions were analyzed by SDS-PAGE and Coomassie staining and found to be about 90% (USP7, both wild-type and inactive mutant), 45% (TIN2), 80% (POT1), and 60%/15% (STN1-CTC1) pure. Telomerase was purified from HEK293T cells to high purity as described (28).

##### Immunofluorescence Analysis

Immunofluorescence analysis was performed as described (29) except that prior to fixation cells were pre-extracted by incubation for 7 min at 4 °C in 20 mm Hepes/KOH, pH 7.9, 50 mm NaCl, 2 mm MgCl_2_, 0.5% Triton X-100, 300 mm sucrose.

##### Chromatin Immunoprecipitation

Chromatin immunoprecipitation assays were performed as described previously (18) with the following modifications. For immunoprecipitations, 20 μl of anti-FLAG affinity gel was added to extracts followed by overnight incubation at 4 °C. Telomeric DNA was detected on a dot blot with a probe generated as follows. A template mixture of 1–5-kb-long telomeric DNA fragments was synthesized by ligating double-stranded telomeric DNA oligonucleotides ((TTAGGG)_5_ and (CCCTAA)_5_) amplified by PCR. The probe was randomly labeled with [α-^32^P]dCTP for detection of the TTAGGG strand. Short interspersed elements of the Alu family were detected as described (18).

##### Binding Assays

To 25 μl of anti-HA-agarose beads with purified ubiquitinated TPP1 in 100 μl of binding buffer (25 mm Tris/HCl, pH 8.0, 150 mm NaCl, 2 mm MgCl_2_) supplemented with 10 mm DTT, either purified USP7(WT)-3xFLAG or buffer control was added followed by incubation for 2 h on a ThermoShaker (interval shaking at 1200 rpm at 37 °C). Samples were washed twice with binding buffer, and the supernatant was removed. 120 μl of binding buffer supplemented with 0.2 μg/μl bovine serum albumin was added followed by incubation for 30 min on a ThermoShaker (interval shaking at 1200 rpm at 4 °C). The respective purified proteins were then added followed by incubation for 2 h on a ThermoShaker (interval shaking at 1200 rpm at 4 °C). Samples were washed three times with binding buffer, and bound proteins were eluted from the beads with SDS-PAGE sample buffer without reducing agent for 5 min at 50 °C. DTT was added after the elution to a final concentration of 50 mm.

##### Half-life Analyses

Cells stably expressing TPP1–3xHA constructs were treated with solvent (DMSO) or MG132 for 2 h followed by addition of cycloheximide to a final concentration of 100 μg/ml. Cells were then directly resuspended in SDS-PAGE sample buffer and boiled for 8 min at 96 °C followed by SDS-PAGE and Western blot analysis.

## RESULTS

### 

#### 

##### TPP1 Interacts with the Deubiquitinase USP7

**FIGURE 1.**
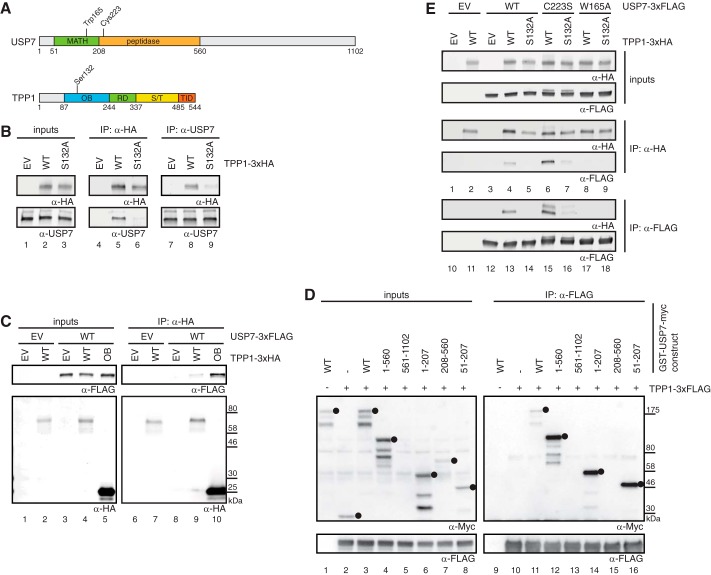
**TPP1 interacts with USP7.** *A*, schematic depiction of USP7 and TPP1 domain organizations. *RD*, recruitment domain for interaction with POT1; *S/T*, Ser/Thr-rich domain; *TID*, TIN2 interaction domain. *B*, cells were transfected with the indicated TPP1 constructs. 48 h post-transfection, extracts were prepared (*lanes 1–3*) followed by anti-HA (*lanes 4–6*) or anti-USP7 (*lanes 7–9*) immunoprecipitation (*IP*) and Western blot analysis. TPP1(WT)-3xHA is co-immunoprecipitated with endogenous USP7, and the interaction is weakened by an S132A mutation in TPP1. *C*, cells were co-transfected with the indicated USP7 and TPP1 constructs. 48 h post-transfection, extracts were prepared (*lanes 1–5*) followed by anti-HA immunoprecipitation (*lanes 6–10*) and Western blot analysis. The OB domain of TPP1 is sufficient for the interaction with USP7. *D*, cells were co-transfected with the indicated USP7 and TPP1 constructs. 48 h post-transfection, extracts were prepared (*lanes 1–8*) followed by anti-FLAG immunoprecipitation (*lanes 9–16*) and Western blot analysis. *Dots* indicate the GST-tagged USP7 fragments. Note that fragment USP7(561–1102) was not expressed in cells. The MATH domain of USP7 mediates its interaction with TPP1. *E*, cells were co-transfected with the indicated USP7 and TPP1 constructs. 48 h post-transfection, extracts were prepared (*inputs*) followed by anti-HA (*lanes 1–9*) or -FLAG (*lanes 10–18*) immunoprecipitation and Western blot analysis. Compared with USP7(WT), USP7(C223S) shows a stronger interaction and USP7(W165A) shows no interaction with TPP1. *EV*, empty vector.

Telomerase activity is regulated at many different levels. One potential target of telomerase regulation is its recruitment to telomeres, which depends on the OB domain of TPP1 (18–21). To identify putative novel interactors of the OB domain of TPP1, we screened a normalized HeLa S3 cell library using a LexA-based yeast two-hybrid system (30). This screen identified USP7 as a factor that interacts with the OB domain of TPP1. USP7, also known as HAUSP, is a Cys-dependent deubiquitinating enzyme with many different substrates and therefore functions in several cellular processes (31) (Fig. 1*A*). Its most prominent role is in the regulation of the p53-HDM2 pathway because USP7 deubiquitinates both p53 and HDM2 (32, 33).

To confirm the yeast two-hybrid screen result, we used a co-immunoprecipitation approach in HEK293T cells and observed that endogenous USP7 co-precipitated ectopically expressed TPP1–3xHA (Fig. 1*B*, *lane 8*) and vice versa (Fig. 1*B*, *lane 5*). The OB domain of TPP1 was used as a bait protein in the yeast two-hybrid screen, and correspondingly, the OB domain of TPP1–3xHA was sufficient to precipitate USP7–3xFLAG (Fig. 1*C*, *lane 10*). Based on structural studies, it was suggested that USP7 binds an (aliphatic/Glu)-Gly-(aliphatic/Glu)-Ser or a (Pro/Ala)-*X-X*-Ser peptide motif in its substrates (Refs. 34 and 35, respectively), and mutation of Ser at the last position of this consensus to Ala would reduce the USP7-substrate interaction. We found two such motifs in the TPP1(OB) domain, amino acids 129–132 (AGPS) and 250–253 (AGLS). Whereas TPP1(S253A)-3xHA interacted with USP7 as strongly as TPP1(WT)-3xHA,^4^ the TPP1(S132A) mutant showed strongly reduced co-immunoprecipitation with USP7 (Fig. 1*B*, compare *lane 5* with *lane 6* and *lane 8* with *lane 9*), indicating that TPP1 may be recognized by USP7 as a substrate. We next addressed which domain of USP7 interacts with TPP1 using a series of GST-tagged USP7 fragments. Whereas the C-terminal part of USP7 was not expressed in cells, the N-terminal part, more specifically the substrate-binding domain (meprin and TRAF (tumor necrosis factor receptor-associated factor) homology (MATH) domain), of USP7 was sufficient to co-precipitate TPP1–3xFLAG (Fig. 1*D*, *lane 16*). Mutation of Trp-165 to Ala in the MATH domain of USP7 has been described to reduce the interaction between USP7 and several of its known substrates (35). Interestingly, USP7(W165A)-3xFLAG did not co-precipitate with TPP1–3xHA (Fig. 1*E*, compare *lane 4* with *lane 8*) and vice versa (Fig. 1*E*, compare *lane 13* with *lane 17*), again raising the possibility that TPP1 is a USP7 substrate. Mutation of Cys-223 to Ser abrogates the enzymatic activity of USP7 (36). In our co-immunoprecipitation assay, USP7(C223S)-3xFLAG displayed a stronger interaction with TPP1–3xHA than USP7(WT)-3xFLAG (Fig. 1*E*, compare *lane 4* with *lane 6* and *lane 13* with *lane 15*), which may be explained by enzyme-substrate trapping. Intriguingly, when USP7(C223S)-3xFLAG was used as a bait protein, it co-precipitated TPP1–3xHA at its usual molecular weight but also a slower migrating form that might correspond to monoubiquitinated TPP1 (Fig. 1*E*, *lane 15*). Together, these data confirm USP7 as a novel interactor of TPP1 and suggest that TPP1 is a USP7 substrate.

##### TPP1 Is Ubiquitinated

**FIGURE 2.**
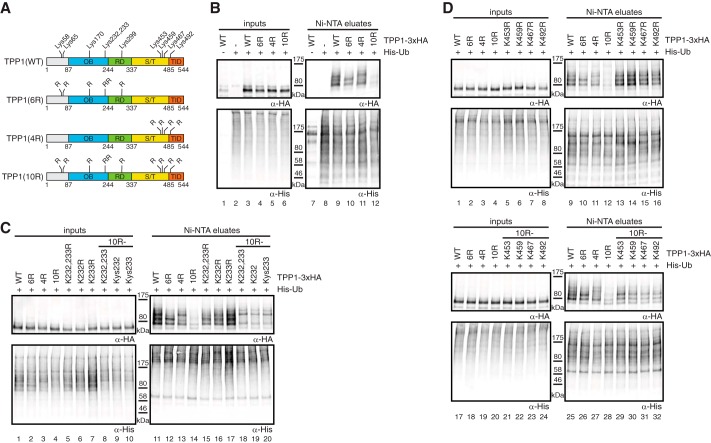
**TPP1 is ubiquitinated at several Lys residues.** *A*, scheme indicating the 10 Lys residues of TPP1(WT) of which the six most N-terminal are mutated to Arg in TPP1(6R), the four most C-terminal are mutated in TPP1(4R), and all 10 are mutated in TPP1(10R). *RD*, recruitment domain for interaction with POT1; *S/T*, Ser/Thr-rich domain; *TID*, TIN2 interaction domain. *B*, cells were co-transfected with His-ubiquitin (*Ub*) and the indicated TPP1–3xHA constructs. 46 h post-transfection, cells were treated with 10 μm MG132. After 2 h, cells were harvested followed by urea extraction (*lanes 1–6*), Ni-NTA binding (*lanes 7–12*), and Western blot analysis. TPP1–3xHA is ubiquitinated, and its ubiquitination pattern changes for both the TPP1(6R) and TPP1(4R) mutants. *C*, ubiquitination assays were performed as in *B* using TPP1–3xHA constructs containing Lys to Arg mutations or Lys residues only at the positions indicated. Lys-232 and Lys-233 of TPP1–3xHA are ubiquitinated. *D*, ubiquitination assays were performed as in *B* using TPP1 constructs containing Lys to Arg mutations or Lys residues only at the positions indicated. Any of the C-terminal four Lys residues of TPP1–3xHA can be ubiquitinated.

If USP7 recognizes TPP1 as a substrate, TPP1 should be ubiquitinated. TPP1 contains 10 Lys residues and thus 10 potential ubiquitination sites (Fig. 2*A*). We tested for TPP1 ubiquitination by co-expressing TPP1–3xHA with a His-tagged ubiquitin construct in HEK293T cells and enriching ubiquitinated proteins using Ni-NTA binding (Fig. 2*B*). TPP1–3xHA was specifically enriched on NTA upon co-expression with His-ubiquitin (Fig. 2*B*, compare *lanes 7* and *9*), showing that TPP1–3xHA is indeed ubiquitinated. Rather than long polyubiquitin chains, only a few ubiquitin moieties appeared to be conjugated to TPP1 even though cells were treated with the proteasome inhibitor MG132. We next tested a series of mutants of TPP1 in which the first six (6R), the last four (4R), or all 10 Lys residues (10R) were mutated to Arg (Fig. 2*A*) to try to dissect where TPP1 might be ubiquitinated and to control for specificity. Indeed, as expected, TPP1(10R)-3xHA was not ubiquitinated and displayed only background signal (Fig. 2*B*, *lane 12*). Interestingly, both TPP1(6R)-3xHA and TPP1(4R)-3xHA were ubiquitinated (Fig. 2*B*, *lanes 10* and *11*), and for both constructs, the ubiquitination pattern was reproducibly distinct from the pattern of TPP1(WT)-3xHA, indicating that TPP1 can be ubiquitinated at several Lys residues. We tested a series of Lys mutants of TPP1 to determine which of its Lys residues can undergo ubiquitination (Fig. 2, *C* and *D*). Single mutation of either Lys-232 or Lys-233 to Arg did not change the ubiquitination pattern of TPP1–3xHA (Fig. 2*C*, compare *lane 11* with *lanes 16* and *17*), whereas TPP1(K232R,K233R)-3xHA displayed a very similar pattern as TPP1(6R)-3xHA (Fig. 2*C*, *lanes 12* and *15*). Conversely, reintroduction of either Lys-232 or Lys-233 into TPP1(10R)-3xHA led to ubiquitination of TPP1 reminiscent of TPP1(4R)-3xHA (Fig. 2*C*, compare *lane 13* with *lanes 18–20*). This indicates that in the OB domain of TPP1 Lys-232 and Lys-233 are ubiquitinated in a redundant manner. The discrete band pattern indicates addition of three to four ubiquitin moieties. In the C-terminal region of TPP1, mutation of single Lys residues to Arg did not change the ubiquitination pattern of TPP1–3xHA, and reintroduction of single Lys residues into TPP1(10R)-3xHA at positions 453, 459, 467, or 492 restored TPP1–3xHA ubiquitination (Fig. 2*D*, *lanes 13–16* and *29–32*). Therefore, TPP1 ubiquitination at the C terminus appears not to be restricted to one Lys residue. In summary, these data show that TPP1 is ubiquitinated at several residues and that TPP1 does not carry long polyubiquitin chains.

##### TPP1 Is Deubiquitinated by USP7

**FIGURE 3.**
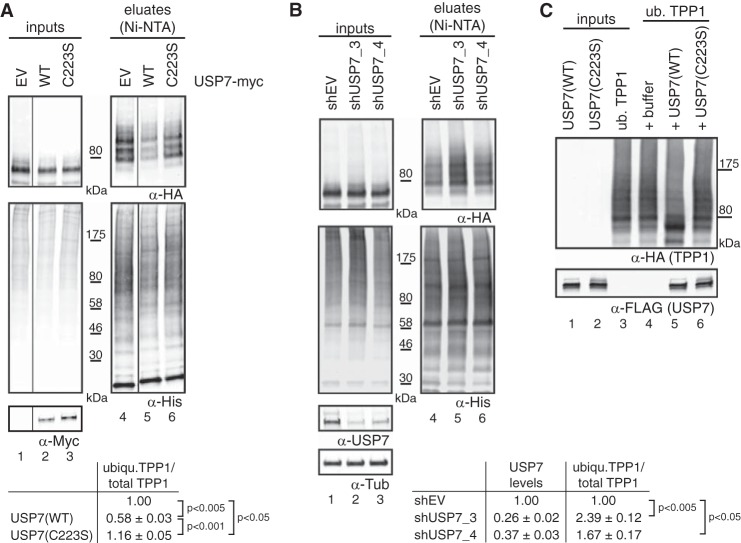
**TPP1 is deubiquitinated by USP7.** *A*, cells were co-transfected with His-ubiquitin, TPP1–3xHA, and the indicated USP7 constructs. 48 h post-transfection, cells were harvested followed by urea extraction (*lanes 1–3*), Ni-NTA binding (*lanes 4–6*), and Western blot analysis. *Vertical lines* separate different parts of the same gel. Quantification of ubiquitinated (*ubiqu*.) TPP1 over total TPP1 from three independent experiments reveals that USP7(WT) but not inactive USP7(C223S) reduces TPP1–3xHA ubiquitination. Average values, standard deviations, and *p* values as determined by *t* test are indicated. *B*, cells were transfected with the indicated shRNA constructs. Puromycin selection was started 20 h post-transfection and continued throughout the experiment. 48 h after shRNA transfections, cells were transfected with His-ubiquitin and TPP1–3xHA. After a further 48 h, cells were harvested followed by urea extraction (*lanes 1–3*), Ni-NTA binding (*lanes 4–6*), and Western blot analysis. Quantification from three independent experiments shows that USP7 depletion increases TPP1–3xHA ubiquitination. Average values, standard deviations, and *p* values as determined by *t* tests are indicated. *C*, ubiquitinated (*ub.*) TPP1 was purified as described under “Experimental Procedures” (*lane 3*) and incubated with either buffer (*lane 4*), purified USP7(WT) (*lane 5*), or USP7(C223S) (*lane 6*). Western blot analysis reveals that USP7(WT) but not USP7(C223S) deubiquitinates TPP1–3xHA *in vitro. EV*, empty vector; *Tub*, tubulin.

Because TPP1 is ubiquitinated and the co-immunoprecipitation data suggested that it is a USP7 substrate, we next tested whether USP7 indeed deubiquitinates TPP1. First, we repeated the ubiquitination assay from HEK293T cells that, in addition to the reporter constructs, expressed either USP7(WT)-myc or the inactive USP7(C223S)-myc (Fig. 3*A*). Quantification of ubiquitinated TPP1 over total TPP1 revealed that USP7(WT)-myc reproducibly reduced the levels of TPP1–3xHA ubiquitination (Fig. 3*A*, compare *lane 5* with *lanes 4* and *6*). USP7(C223S)-myc in contrast led to slightly increased ubiquitination and therefore had a minor dominant negative effect on TPP1–3xHA ubiquitination levels. To further determine the effects of USP7 on TPP1 ubiquitination in cells, we depleted USP7 with two different shRNAs and verified USP7 depletion by Western blotting with anti-USP7 antibodies (Fig. 3*B*, *lanes 1–3*). Both shRNAs led to increased TPP1–3xHA ubiquitination with the stronger effect linked to the more efficient shRNA (Fig. 3*B*, compare *lanes 4–6*). Together, these data indicate that USP7 deubiquitinates TPP1. To corroborate this finding, we set up an *in vitro* reaction using purified ubiquitinated TPP1–3xHA, which we incubated with partially purified USP7(WT)-3xFLAG or inactive USP7(C223S)-3xFLAG. Addition of USP7(WT)-3xFLAG but not USP7(C223S)-3xFLAG reduced TPP1 ubiquitination (Fig. 3*C*, *lanes 5* and *6*), confirming that TPP1 is a USP7 substrate. Therefore, both *in vivo* and *in vitro* evidence indicates that TPP1 is deubiquitinated by USP7.

##### Ubiquitination-deficient TPP1 Mutants Localize to the Telomere

**FIGURE 4.**
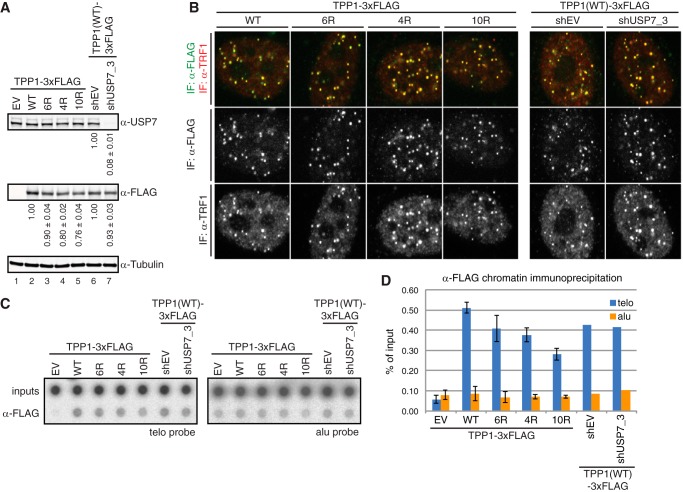
**Ubiquitination-deficient TPP1 mutants localize to telomeres.** *A*, Western blot analysis of cell lines stably expressing TPP1(WT)-3xHA or ubiquitination-deficient mutants. Relative, normalized levels of TPP1–3xHA constructs and USP7 are indicated below the respective blots, indicating similar expression levels for the TPP1–3xHA constructs and efficient depletion of USP7 by shRNA. Average values and standard deviations are indicated. *B*, immunostaining of cells from *A* for TPP1–3xHA constructs and TRF1. TPP1(WT)-3xHA and ubiquitination-deficient mutants co-localize with TRF1. USP7 depletion does not change telomere association of TPP1(WT)-3xHA as observed in several independent experiments. *C*, chromatin immunoprecipitation of TPP1–3xHA constructs from cells in *A. D*, quantification of *C*. TPP1(WT)-3xHA and ubiquitination-deficient mutants precipitate telomeric DNA. Average values and standard deviations (*error bars*) are indicated. USP7 depletion does not change telomere association of TPP1(WT)-3xHA. Only one ChIP quantification was performed for USP7 depletion because the immunostaining in *B* consistently showed unchanged TPP1(WT)-3xHA telomere association in the absence of USP7. *EV*, empty vector; *IF*, immunofluorescence.

Ubiquitination of TPP1 might be used to regulate its localization and function. Interestingly, studies in mouse cells have shown that Tpp1 needs to be ubiquitinated to associate with telomeres because mutation of Lys-233 in mouse Tpp1 led to loss of co-localization with telomeres (23). To analyze the localization of ubiquitination-deficient TPP1 mutants in human cells, HeLa cell lines were established that stably overexpress the respective constructs (Fig. 4*A*). Localization of the TPP1 mutants was analyzed by co-immunostaining for their 3xFLAG tag and TRF1 as a telomeric marker. As expected, TPP1(WT)-3xFLAG co-localized with TRF1, indicating its telomeric localization (Fig. 4*B*). Surprisingly, the same was true for TPP1(6R)-, TPP1(4R)-, and TPP1(10R)-3xFLAG. Chromatin immunoprecipitation experiments confirmed these findings because not only TPP1(WT) but also the ubiquitination-deficient mutants precipitated telomeric DNA with comparable efficiencies (Fig. 4, *C* and *D*). Therefore, in contrast to the situation in mouse cells, ubiquitination of human TPP1 is not required for its localization to telomeres.

To test whether ubiquitination prevents TPP1 localization to telomeres, we depleted USP7 by shRNA transfection in the cells stably expressing TPP1(WT)-3xFLAG. Whereas USP7 depletion was efficient (Fig. 4*A*, *lane 7*), TPP1(WT)-3xFLAG telomeric localization did not change as determined by co-staining with TRF1 (Fig. 4*B*) and chromatin immunoprecipitation (Fig. 4, *C* and *D*). Therefore, a mild increase in ubiquitination as observed after USP7 depletion (Fig. 3*B*) does not prevent telomere localization of TPP1.

##### Ubiquitination-deficient TPP1 Mutants Retain Interactions with TIN2, POT1, Telomerase, and the CST Complex

**FIGURE 5.**
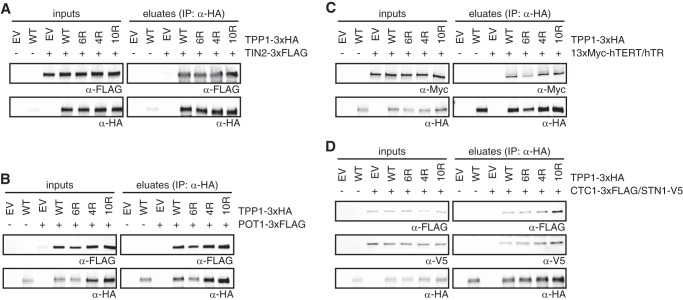
**Ubiquitination-deficient TPP1 mutants are able to interact with TIN2, POT1, CTC1-STN1, and telomerase.** Cells were co-transfected with the indicated constructs. 48 h post-transfection, extracts were prepared followed by anti-HA immunoprecipitation (*IP*) and Western blot analysis. TPP1(WT)-3xHA and ubiquitination-deficient mutants interact with TIN1–3xFLAG (*A*), POT1–3xFLAG (*B*), telomerase (*C*), and CTC1–3xFLAG/STN1-V5 (*D*). *EV*, empty vector.

TPP1 contributes to the integrity of the shelterin complex via its interaction with TIN2 and POT1, which are required to anchor POT1 at telomeres (12–15). To test whether TPP1 ubiquitination is required for its interactions with these two proteins, we performed co-immunoprecipitation experiments from extracts of HEK293T cells co-expressing the different TPP1–3xHA constructs with either TIN2–3xFLAG (Fig. 5*A*) or POT1–3xFLAG (Fig. 5*B*). As expected, TPP1(WT)-3xHA co-precipitated both TIN2–3xFLAG and POT1–3xFLAG. The same result was observed for all ubiquitination-deficient mutants of TPP1, indicating that ubiquitination does not contribute to TIN2 and POT1 binding. TPP1 is also known to interact with telomerase, thereby contributing to telomerase recruitment and stimulation (18–21). Therefore we tested whether this interaction might require TPP1 ubiquitination. However, as for TIN2 and POT1, the co-immunoprecipitation assays showed that not only TPP1(WT)-3xHA but also the ubiquitination-deficient mutants co-precipitated telomerase (Fig. 5*C*). Finally, recent data showed that the CST complex interacts with TPP1-POT1, and it was proposed that CST inhibits the stimulatory effect of TPP1-POT1 on telomerase (25). We co-expressed CTC1–3xFLAG and STN1-V5 with TPP1(WT)-3xHA or the ubiquitination-deficient mutants and again observed in co-immunoprecipitation assays that TPP1 ubiquitination is not necessary for CTC1-STN1 binding (Fig. 5*D*). Therefore, ubiquitination of TPP1 does not contribute in a detectable manner to its known interactions with TIN2, POT1, telomerase, and the CST complex.

##### Ubiquitinated TPP1 Is Able to Interact with TIN2, POT1, Telomerase, and the CST Complex

**FIGURE 6.**
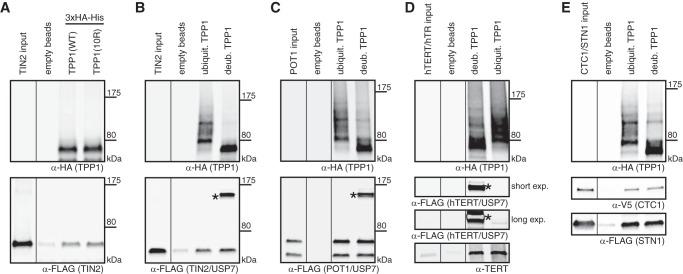
**Ubiquitinated TPP1 is able to interact with TIN2, POT1, CTC1-STN1, and telomerase.** *A*, binding assay using TPP1(WT)- or TPP1(10R)-3xHA-His and TIN2 purified from HEK293T cells as described under “Experimental Procedures” and analyzed by Western blotting. *Vertical lines* separate different parts of the same gel. Both TPP1 constructs are able to interact with TIN2–3xFLAG upon denaturation/refolding during the purification process. Purified ubiquitinated (*ubiquit.*) TPP1–3xHA or *in vitro* deubiquitinated (*deub.*) TPP1–3xHA was incubated in binding assays with TIN2–3xFLAG (*B*), POT1–3xFLAG (*C*), telomerase (*D*), or CTC1-V5/STN1–3xFLAG (*E*) purified from HEK293T cells as described under “Experimental Procedures.” *Vertical lines* separate different parts of the same gel. *Asterisks* indicate USP7–3xFLAG used for deubiquitination. Western blot analysis reveals no difference in binding of ubiquitinated or deubiquitinated TPP1–3xHA to the factors tested.

Analysis of the ubiquitination-deficient mutants of TPP1 allowed determination of whether ubiquitination of TPP1 is required for its functions. However, these mutants cannot be used to analyze whether ubiquitination prevents TPP1 functions. *In vivo* analysis of ubiquitinated TPP1 is difficult because only a low percentage of the protein is ubiquitinated (see Fig. 2*B*, *lane 3*). We tested whether TPP1 ubiquitination is increased in a variety of conditions but did not observe a significant change of TPP1 ubiquitination along the cell cycle or upon telomeric DNA damage.^4^ Furthermore, depletion of USP7 resulted in only a 2-fold increase in TPP1 ubiquitination (Fig. 3*B*). Therefore, to address whether ubiquitination prevents TPP1 functions, we performed a series of *in vitro* binding assays with ubiquitinated TPP1. We purified TPP1 specifically in its ubiquitinated form from cells co-expressing TPP1–3xHA and His-ubiquitin using a two-step procedure via Ni-NTA-agarose and anti-HA immunoprecipitation. This procedure, however, included denaturation and refolding steps. To test whether the refolding process worked, TPP1(WT) and TPP1(10R) were purified as 3xHA-His-tagged proteins via the same two-step procedure. Both proteins were then incubated with partially purified TIN2 in a binding assay and were able to bind TIN2 (Fig. 6*A*). Thus the refolding process must be at least partially successful. Next, we compared the ability of purified ubiquitinated TPP1 with or without USP7-mediated *in vitro* deubiquitination to bind to TIN2 (Fig. 6*B*). Both ubiquitinated and deubiquitinated TPP1 did not differ in their ability to interact with TIN2. Similarly, both versions of TPP1 were able to bind the same amount of POT1 (Fig. 6*C*), telomerase (Fig. 6*D*), and CTC1-V5/STN1–3xFLAG (Fig. 6*E*). Therefore, ubiquitination of TPP1 does not interfere with its known interactions with TIN2, POT1, telomerase, and the CST complex. In summary, we have not been able to detect a role for ubiquitination in regulating TPP1 interactions.

##### TPP1 Is Degraded in a Proteasome-dependent Manner

**FIGURE 7.**
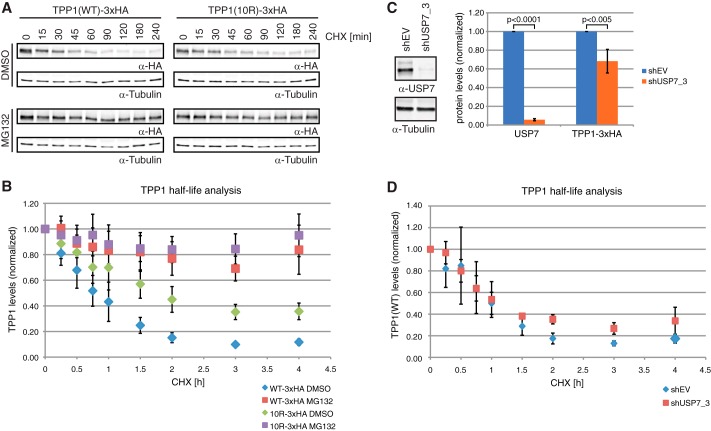
**TPP1 is degraded in a proteasome-dependent manner, but its half-life is not changed upon USP7 depletion.** *A*, cells stably expressing TPP1(WT)- or TPP1(10R)-3xHA were treated with cycloheximide (*CHX*) and MG132 or solvent (DMSO). TPP1 protein levels were analyzed by Western blotting for the indicated time points. *B*, quantification of six experiments as in *A*. TPP1(WT) half-life is prolonged by MG132 treatment, indicating proteasome-dependent degradation of TPP1(WT)-3xHA. The half-life of TPP1(10R)-3xHA is longer than of TPP1(WT)-3xHA but is still further increased by MG132 treatment. Average values and standard deviations (*error bars*) are indicated. *C*, cells stably expressing TPP1(WT)-3xHA were depleted for USP7. Western blot analysis and quantification from five independent experiments are shown. Average values, standard deviations (*error bars*), and *p* values as determined by *t* test are indicated. Efficient USP7 depletion slightly reduces TPP1–3xHA levels. *D*, cells from *C* were used for half-life analysis as in *A*. Quantification from five independent experiments is shown. Average values and standard deviations (*error bars*) are indicated. Efficient USP7 depletion does not significantly change TPP1–3xHA half-life. *EV*, empty vector.

Besides regulating protein function and localization, ubiquitination, in particular polyubiquitination, is used by cells to trigger proteasome-dependent degradation. To investigate whether TPP1 is degraded via the proteasome, we determined the half-life of TPP1 upon proteasome inhibition. Cells stably expressing TPP1(WT)- or TPP1(10R)-3xHA were treated with solvent or the proteasome inhibitor MG132 followed by cycloheximide addition to block protein synthesis. Analysis of TPP1(WT)-3xHA protein levels over a time course of 4 h revealed that its half-life, about 45 min, is relatively short (Fig. 7, *A* and *B*). Inhibition of the proteasome completely stabilized TPP1(WT)-3xHA, indicating that TPP1–3xHA is indeed degraded in a proteasome-dependent manner. TPP1(10R)-3xHA is more stable than TPP1(WT)-3xHA as expected because it cannot undergo ubiquitination. However, this mutant was still further stabilized by MG132 treatment, indicating that there is also an indirect contribution of the proteasome to TPP1 stability (see “Discussion”).

To determine whether USP7 is involved in regulation of TPP1 levels via the proteasome, we repeated the analysis of TPP1(WT)-3xHA half-life in control cells or cells depleted for USP7. USP7 depletion was very efficient and indeed led to a slight reduction in TPP1(WT)-3xHA levels (Fig. 7*C*). However, TPP1(WT)-3xHA half-life appeared unaffected by the presence or absence of USP7 (Fig. 7*D*). Together, these data clearly show that TPP1 protein levels are regulated by the proteasome. USP7 depletion did not affect this process, but USP7 might act in a redundant manner with other deubiquitinases to stabilize TPP1.

## DISCUSSION

Shelterin proteins are important players in telomere biology and essential for preventing DNA damage repair at chromosome ends and in regulating telomere elongation. TPP1 has an important role in maintaining shelterin integrity via its interactions with TIN2 and POT1. This role appears to account for its negative control of telomerase as it places the single strand DNA-binding protein POT1 at telomere ends and prevents access of telomerase to its substrate. However, it has become clear that TPP1 is also necessary as a positive regulator of telomerase by mediating its recruitment to telomeres and, together with POT1, stimulating telomerase activity and processivity. Furthermore, telomerase activity is controlled for instance along the cell cycle with telomere elongation occurring in S phase or at the level of individual telomeres with elongation of short telomeres rather than long telomeres. Shelterin proteins, especially TPP1 as a recruiting factor for telomerase, are prime candidates to be involved in these aspects of telomere biology. These distinct roles and other aspects of TPP1 biology are likely influenced by post-translational modifications as exemplified by the proposed contribution of TPP1 phosphorylation to telomerase activation (22) or the ubiquitination-dependent telomere localization of Tpp1 in mouse cells (23).

In this study, we have identified the deubiquitinating enzyme USP7 as an interaction partner of human TPP1, suggesting a role for ubiquitination in TPP1 biology. We observed that TPP1 is ubiquitinated, and it is deubiquitinated by USP7. Our attempts to determine which E3 ligase(s) might ubiquitinate TPP1 were not successful, so it remains unclear how TPP1 is ubiquitinated. We detected TPP1 ubiquitination at multiple Lys residues in its OB domain and C-terminal region. Intriguingly, the Lys-232 and Lys-233 ubiquitination sites in the TPP1 OB domain were also found to be ubiquitinated in mouse Tpp1 (23), and Lys-233 of mouse Tpp1 was critical for its telomere localization. Strikingly, however, whereas these ubiquitination sites are conserved, the function of the corresponding ubiquitination is not. A mutant of TPP1 in which all 10 Lys residues were mutated to Arg still localized to the telomeres. Therefore, human TPP1 and mouse Tpp1 appear to be targeted to telomeres in distinct ways. Fundamental differences in human and mouse shelterin components are not unprecedented. For example, whereas human telomeres have one POT1 protein, mouse telomeres require two distinct Pot1 proteins (37).

**FIGURE 8.**
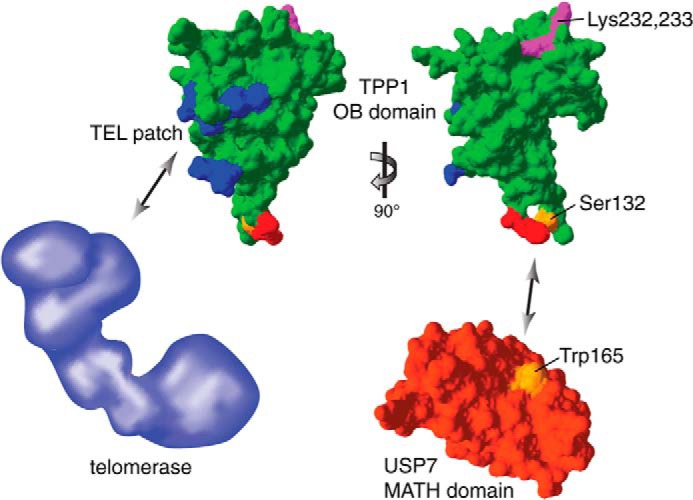
**Structural representation of the TPP1(OB) domain interactions and ubiquitination sites.** The structure of the TPP1(OB) domain is shown in *green* (Protein Data Bank code 2I46) with the TEL patch region in *blue*, the USP7 consensus motif in *red* (Ser-132 in *orange*), and the ubiquitination sites Lys-232 and Lys-233 in *magenta*. The structure of the MATH domain of USP7 is shown in *red* (Protein Data Bank code 1YZE) with Trp-165 in *orange*. Structure images were generated with Swiss-Pdb Viewer (38). The schematic representation of telomerase in *blue* is not drawn to scale. The TPP1(OB) domain interacts with telomerase and USP7 via distinct surfaces. Although the ubiquitination sites of the TPP1(OB) domain appear to be on a different surface than the USP7 consensus motif, they are likely accessible for the catalytic peptidase domain of USP7, allowing USP7 to deubiquitinate TPP1.

We searched for further aspects of human TPP1 function that may be affected positively or negatively by ubiquitination. Our data did not identify ubiquitination events that contribute to the regulation of protein-protein interactions involving TPP1 or properties of TPP1 that are regulated by USP7-mediated deubiquitination. However, our analysis may have missed subtle effects of USP7 on TPP1 function or the regulation of cell cycle-specific interactions, and it remains possible that so far unknown interactions and functions of TPP1 are controlled by ubiquitination and USP7. Moreover, because of the presence of multiple ubiquitination sites in TPP1, we may have been limited in detecting regulatory monoubiquitinations on a particular TPP1 Lys residue. Interestingly, one of the conserved Lys residues of TPP1 is Lys-170 in its OB domain and placed in the TEL patch that mediates TPP1-telomerase interaction and telomerase stimulation by TPP1 (19–21). It is tempting to speculate that ubiquitination of Lys-170 would abrogate a TPP1-telomerase interaction and allow regulation of telomerase recruitment, but we were unable to detect such a ubiquitination event.^4^ Prior to this study, only telomerase was known to physically interact with the OB fold of TPP1. Our work identifies that the MATH domain of USP7 binds to the OB fold of TPP1. Besides Trp-165 of USP7 that is placed along the peptide-binding groove of USP7 that allows substrate recognition, we identify Ser-132 in TPP1 as critical for this interaction (Fig. 8). The USP7 recognition motif with Ser-132 forms a surface-exposed loop in the TPP1(OB) domain, suggesting that it is readily accessible for USP7. Interestingly, USP7-TPP1 interaction was equally detected with wild-type and ubiquitination-defective TPP1,^4^ indicating that USP7 can bind ubiquitinated and deubiquitinated TPP1. As discussed above, telomerase also interacts with the OB fold of TPP1, and its binding surface (referred to as the TEL patch) was characterized through mutations (Fig. 8). TPP1 Ser-132, which is critical for MATH domain binding, seems slightly remote from the TEL patch, indicating that USP7 and telomerase recognize different surfaces on TPP1. However, the distance between the TEL patch and the loop containing Ser-132 is rather short, and considering that both USP7 and telomerase are large molecules, it is possible that these two factors affect the binding of each other to TPP1. It will be interesting to compare the structures of interacting polypeptides and determine whether and how the two proteins compete with each other for TPP1 binding *in vivo*.

Our study also addresses the question of how ubiquitination affects and regulates TPP1 protein levels. Whereas TPP1(WT) half-life is less than 1 h in control cells, it is much prolonged upon proteasome inhibition. Intriguingly, the ubiquitination-deficient TPP1(10R) mutant is more stable than TPP1(WT) with a roughly 2-fold extended half-life. However, the half-life of TPP1(10R) is also strikingly extended by MG132 treatment, indicating that there is an indirect contribution of the proteasome to TPP1 stability. Possibly a binding partner of TPP1 that affects its stability is, in turn, also degraded by the proteasome. Although depletion of USP7 led to slightly reduced TPP1 levels, it did not have a clear effect on TPP1 half-life. However, it remains possible that USP7 acts in a redundant manner with other deubiquitinases to counteract proteasome-dependent degradation of TPP1. Via its interactions with POT1 and TIN2, TPP1 is important for the structural integrity of the shelterin complex. It seems obvious that cells should control TPP1 levels because changes in its stoichiometry could affect the shelterin complex by failing to properly localize POT1 or by titrating away TIN2 and POT1. Indeed, TPP1 depletion leads to telomere elongation (12–14), and so does its overexpression (12) although not in all experimental settings (13, 14). Therefore, regulation of TPP1 levels via ubiquitination and proteasome-dependent degradation allows cells to maintain a functional shelterin complex.

## Footnotes

4 I. Zemp and J. Lingner, unpublished observations.

## Footnotes

3 The abbreviations used are:

OB: oligonucleotide/oligosaccharide-binding
TEL: TPP1 glutamine (E)- and leucine (L)-rich
CST: CTC1-STN1-TEN1
MATH: meprin and TRAF (tumor necrosis factor receptor-associated factor) homology
Ni-NTA: nickel-nitriloacetic acid
hTR: human telomerase RNA
hTERT: human telomerase reverse transcriptase.
